# Lemierre Syndrome Following Dental Extraction: A Case Report

**DOI:** 10.7759/cureus.108015

**Published:** 2026-04-30

**Authors:** Yesenia Ortega Flores, Oliver A Colis Arenas, Marian A Rodriguez Carrillo, Magda Karina López Saldaña, Miguel Ángel Galindo López

**Affiliations:** 1 Department of Education and Research, Centenario Hospital Miguel Hidalgo, Aguascalientes, MEX; 2 Department of Internal Medicine, Centenario Hospital Miguiel Hidalgo, Aguascalientes, MEX; 3 Department of Internal Medicine, Centenario Hospital Miguel Hialgo, Aguascalientes, MEX; 4 Department of Internal Medicine, Centenario Hospital Miguel Hidalgo, Aguascalientes, MEX

**Keywords:** atypical lemierre syndrome, internal jugular venous thrombosis, ludwig's angina, odontogenic infection, pulmonary septic emboli

## Abstract

Lemierre syndrome is a rare but potentially life-threatening condition characterized by septic thrombophlebitis of the internal jugular vein and hematogenous dissemination of septic emboli. Although classically associated with oropharyngeal infections, atypical presentations from odontogenic sources are increasingly recognized and may complicate early diagnosis. We report the case of a 29-year-old male patient who developed Lemierre syndrome following lower third molar extraction. He presented with right cervical swelling, dysphonia, odynophagia, and cough. Laboratory findings showed leukocytosis with neutrophilia. Imaging studies revealed a deep cervical collection consistent with Ludwig’s angina, extensive thrombosis of the right internal jugular vein with extension to the jugular bulb, sigmoid sinus, and subclavian vein, and multiple bilateral pulmonary lesions suggestive of septic emboli.

The patient underwent surgical drainage and received broad-spectrum antibiotic therapy along with anticoagulation due to the extent of thrombosis. Microbiological cultures were negative, likely influenced by prior antibiotic exposure and the inherent difficulty in isolating anaerobic pathogens such as *Fusobacterium *species. Despite significant radiological involvement, clinical evolution was favorable, without hemodynamic instability or respiratory compromise. Follow-up demonstrated a reduction of pulmonary lesions and eventual resolution of thrombosis.

This case highlights an uncommon odontogenic origin with unusually extensive venous propagation, including intracranial extension, in the setting of a relatively benign systemic course. It underscores the importance of considering Lemierre syndrome in deep cervicofacial infections after dental procedures, even in the absence of a preceding pharyngeal infection, and reinforces the need for early imaging and multidisciplinary management to enable timely diagnosis and appropriate treatment.

## Introduction

Lemierre syndrome is a rare but potentially life-threatening clinical entity that typically arises as a septic complication of head and neck infections. It is characterized by thrombophlebitis of the internal jugular vein, with subsequent hematogenous dissemination of septic emboli [1,2]. It was first described by André Lemierre in 1936 as a severe form of anaerobic septicemia following a pharyngeal infection, thereby establishing the basis for its clinical recognition [1-3].

Although classically associated with oropharyngeal infections such as pharyngitis or tonsillitis, Lemierre syndrome may originate from various head and neck sources, including mastoiditis, sinusitis, deep cervical infections, and odontogenic infections [1,3-5]. This variability in the site of origin reflects the heterogeneity of its clinical presentation and contributes to the lack of uniform diagnostic criteria [3,5-7].

Regarding etiology, *Fusobacterium necrophorum *remains the most frequently implicated microorganism. However, recent series have documented greater microbiological diversity, including other anaerobic and aerobic bacteria, as well as cases with negative cultures, suggesting that the disease does not depend exclusively on a single pathogen and that microbiological isolation may be challenging [1-3,6,8].

From a pathophysiological perspective, the process generally begins with the progression of infection into the deep spaces of the neck, involving the venous system and leading to septic thrombosis formation [1,4,5,7]. Subsequently, infectious emboli may disseminate to distant sites, most commonly affecting the lungs, although other organs, including the central nervous system, may also be involved in more severe cases [1-5,7,9].

Despite its low incidence, estimated at 0.8 to 5.5 cases per million inhabitants, the syndrome predominantly affects previously healthy young adults and continues to represent a diagnostic challenge, particularly in its early stages, when symptoms may be nonspecific [3-5,8,10]. Following a marked decline during the antibiotic era, several authors have reported a resurgence of the syndrome in recent decades, tentatively attributed to more restrictive antibiotic use in upper respiratory tract infections, changes in prescribing practices, reduced rates of tonsillectomy, and improved diagnostic capabilities through anaerobic cultures and imaging [1,3-5,10].

Clinically, the condition typically evolves from an initial infection to systemic manifestations. Neck pain and unilateral swelling may suggest internal jugular vein thrombosis, whereas later stages are dominated by septic embolization, particularly pulmonary involvement with infiltrates, cavitary nodules, pleural effusion, empyema, or lung abscesses [1-5,7]. Although less common, severe neurological and intracranial complications have also been described, including cavernous sinus thrombosis, cerebral venous thrombosis, meningitis, intracranial abscesses, and cerebral infarction, further expanding the clinical spectrum and complicating early diagnosis [1-5,7,9].

Diagnosis is based on a combination of clinical history, imaging findings, and microbiological studies when available [1,3-7]. Contrast-enhanced computed tomography is the most commonly used imaging modality, although other techniques may complement the evaluation depending on the clinical scenario [1,3,4,9].

Treatment is primarily based on prolonged antibiotic therapy with coverage for anaerobic bacteria, and some patients may require surgical intervention [1-5,7,9]. The use of anticoagulation in Lemierre syndrome remains controversial due to the lack of controlled clinical trials and the heterogeneous nature of the available evidence, which is largely derived from case reports and retrospective series [2,4,7]. Current data have not consistently demonstrated a benefit in terms of vascular recanalization or mortality, although some studies suggest a possible reduction in thromboembolic complications in selected contexts [1,7]. In clinical practice, anticoagulation is generally considered on an individual basis, particularly in patients with extension of the thrombus into the cerebral venous sinuses, extensive or bilateral thrombosis, clinical progression despite appropriate antibiotic therapy, or persistent embolic phenomena [1,2,9].

Despite advances in management, Lemierre syndrome may be associated with significant complications and long-term sequelae [3,5,8]. In this context, recognizing atypical presentations, particularly those of non-oropharyngeal origin such as odontogenic infections, is essential to avoid delays in diagnosis. Therefore, case reports remain valuable for illustrating the variability of this entity and reinforcing the need for a high index of clinical suspicion.

## Case presentation

A 29-year-old male patient with no significant past medical history, aside from chronic alcohol use, discontinued one month prior, underwent extraction of the lower right third molar 10 days before admission. Postoperatively, clindamycin 600 mg every 12 hours was prescribed as prophylaxis.

Eight days after the procedure, two days prior to admission, he developed right-sided cervical swelling and coughing episodes, accompanied by subjective fever, which he self-treated with a single dose of ibuprofen. On the day of presentation, he noted progression of cervical swelling and new-onset dysphonia, prompting evaluation in the emergency department.

On admission, the patient was afebrile and presented with an indurated, erythematous, and painful mass, fixed to deep planes, extending from the right mandibular region to the lateral cervical region. He exhibited dysphonia and progressive odynophagia, raising concern for upper airway involvement. Despite preserved respiratory mechanics and oxygen saturation of 94% on room air, these findings prompted a formal airway risk assessment. Cardiac examination revealed regular heart sounds without murmurs. The chest was symmetrical, with adequate respiratory mechanics and well-ventilated lung fields without added sounds. The remainder of the physical examination was unremarkable.

**Figure 1:**
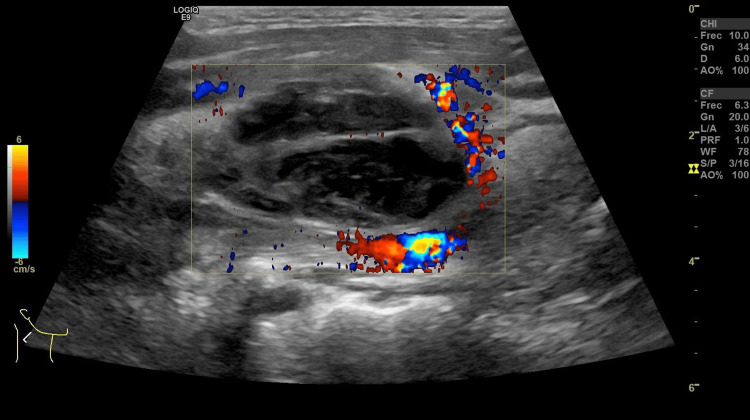
The initial neck ultrasound shows a heterogeneous, predominantly hypoechoic, non-vascularized, septated lesion compatible with Ludwig’s angina.

Initial imaging with anteroposterior and lateral neck radiographs demonstrated displacement of the tracheal air column, suggesting mass effect on the upper airway. A neck ultrasound subsequently revealed a well-defined, heterogeneous hypoechoic collection with peripheral vascularity, associated with adjacent inflammatory changes and reactive lymphadenopathy (Figure 1), consistent with a suppurative process.

Laboratory analysis showed leukocytosis of 21.9 ×10³/µL with neutrophilia (17.69 ×10³/µL) and thrombocytosis (433 ×10³/µL); the remaining parameters were within normal limits. Additional inflammatory markers, including C-reactive protein and procalcitonin, were not obtained due to limited institutional availability.

From admission, the patient underwent close airway monitoring with serial clinical assessments and continuous oxygen saturation surveillance. No signs of impending airway compromise, such as stridor, respiratory distress, or hypoxemia, were observed, and invasive airway management was not required. Adjunctive corticosteroid therapy was administered to reduce airway edema. During the first three days of hospitalization, clindamycin was continued; however, persistent leukocytosis and lack of clinical improvement raised concern for ongoing deep neck infection.

At this stage, the clinical presentation, characterized by rapidly progressive cervical swelling, dysphonia, and odynophagia, raised suspicion for Ludwig’s angina. However, the presence of a localized collection and lymphadenopathy on ultrasound was not entirely consistent with a purely cellulitic process, prompting consideration of alternative diagnoses, including a deep neck space abscess.

**Figure 2:**
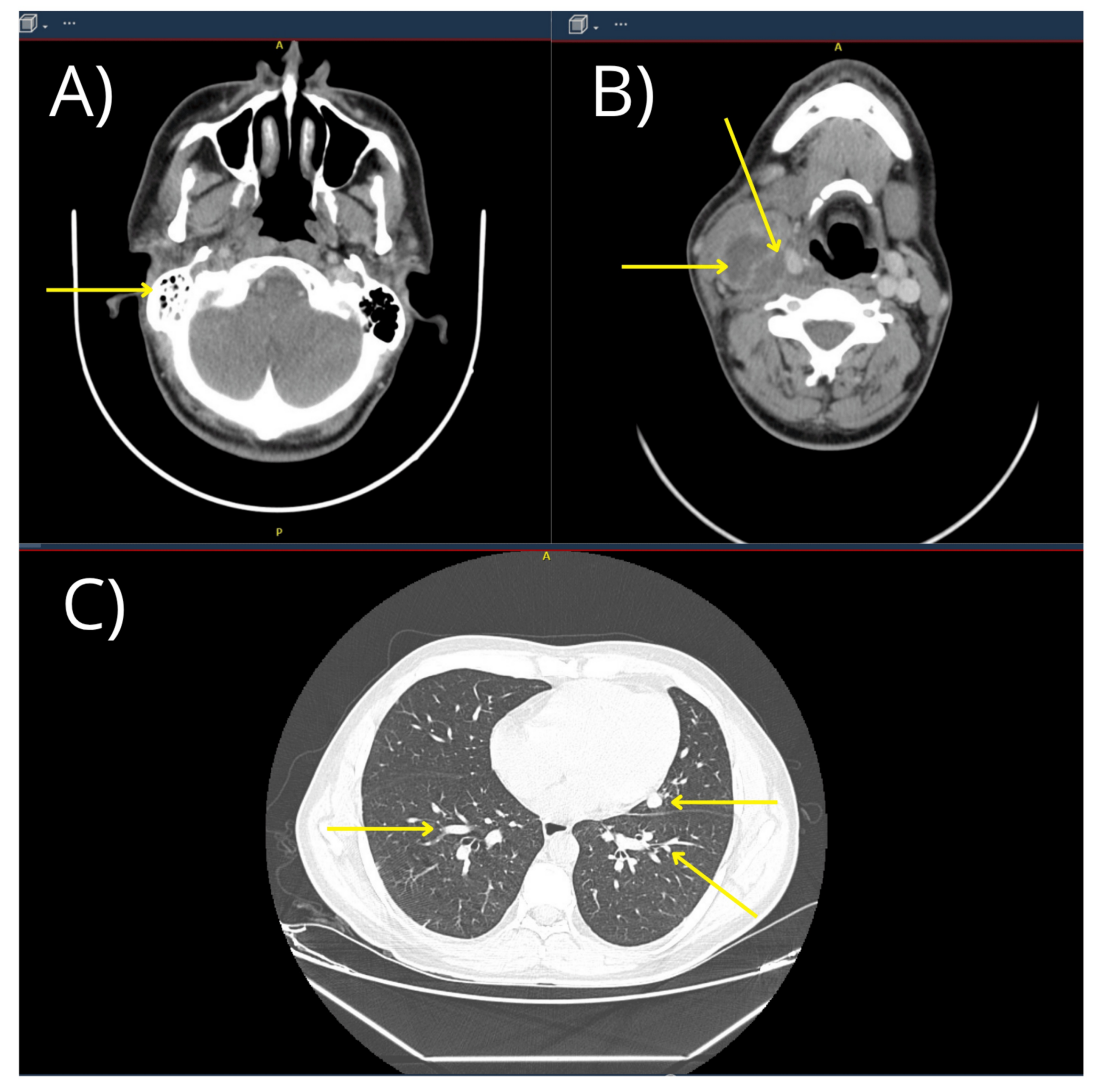
Contrast-enhanced computed tomography of the neck A) Mastoid level showing hypodensity in the right internal jugular vein compatible with venous thrombosis and right otomastoiditis. B) Cricoid cartilage level showing a collection in the right cervical region. C) Pulmonary level showing multiple bilateral peripheral nodular opacities, some demonstrating a probable feeding vessel sign, findings highly suggestive of septic emboli.

Given this diagnostic uncertainty, contrast-enhanced computed tomography of the head, neck, and chest was performed on the second day of hospitalization. Imaging demonstrated a 36 × 56 × 64 mm collection medial to the right sternocleidomastoid muscle, extending from the hyoid to the cricoid cartilage, with leftward displacement of the airway and a patent glottis and subglottis. Additionally, thrombosis of the right internal jugular vein with intracranial extension to the jugular bulb and sigmoid sinus, as well as extension to the subclavian vein, was identified. Associated findings included inflammatory changes of the right mandibular ramus with regional lymphadenopathy, right otomastoiditis, and multiple bilateral pulmonary nodular lesions suggestive of septic emboli (Figure 2). Based on the integration of clinical, laboratory, and imaging findings, a diagnosis of Lemierre syndrome secondary to odontogenic deep neck infection was established.

In light of the extent of thrombus propagation and intracranial involvement, anticoagulation with enoxaparin 1 mg/kg subcutaneously every 12 hours was initiated on the second day of hospitalization. No contraindications to anticoagulation were identified.

On the third day of hospitalization, an otolaryngology consultation was obtained, and surgical drainage was performed via an external cervical approach, yielding abundant greenish purulent material with foul odor. A Penrose drain was placed, and samples were sent for microbiological analysis, including aerobic and anaerobic cultures, which showed no bacterial growth after 48 hours. Blood cultures, obtained after initiation of antibiotic therapy, were likewise negative, possibly influenced by prior clindamycin exposure.

Given the persistence of leukocytosis, concern for anaerobic pathogens, and imaging evidence of a deep neck space abscess, antimicrobial therapy was escalated to intravenous piperacillin-tazobactam, which was continued for 14 days. Upon clinical improvement, the patient was transitioned to oral amoxicillin/clavulanic acid (500 mg every eight hours) for an additional four weeks.

Two weeks after discharge, flexible laryngoscopy demonstrated right vocal cord involvement with paramedian paresis and compensatory mobility of the left vocal cord. The patient subsequently underwent interdisciplinary follow-up, including rehabilitation, maxillofacial surgery, phoniatrics, internal medicine, infectious diseases, angiology, and otolaryngology.

**Figure 3:**
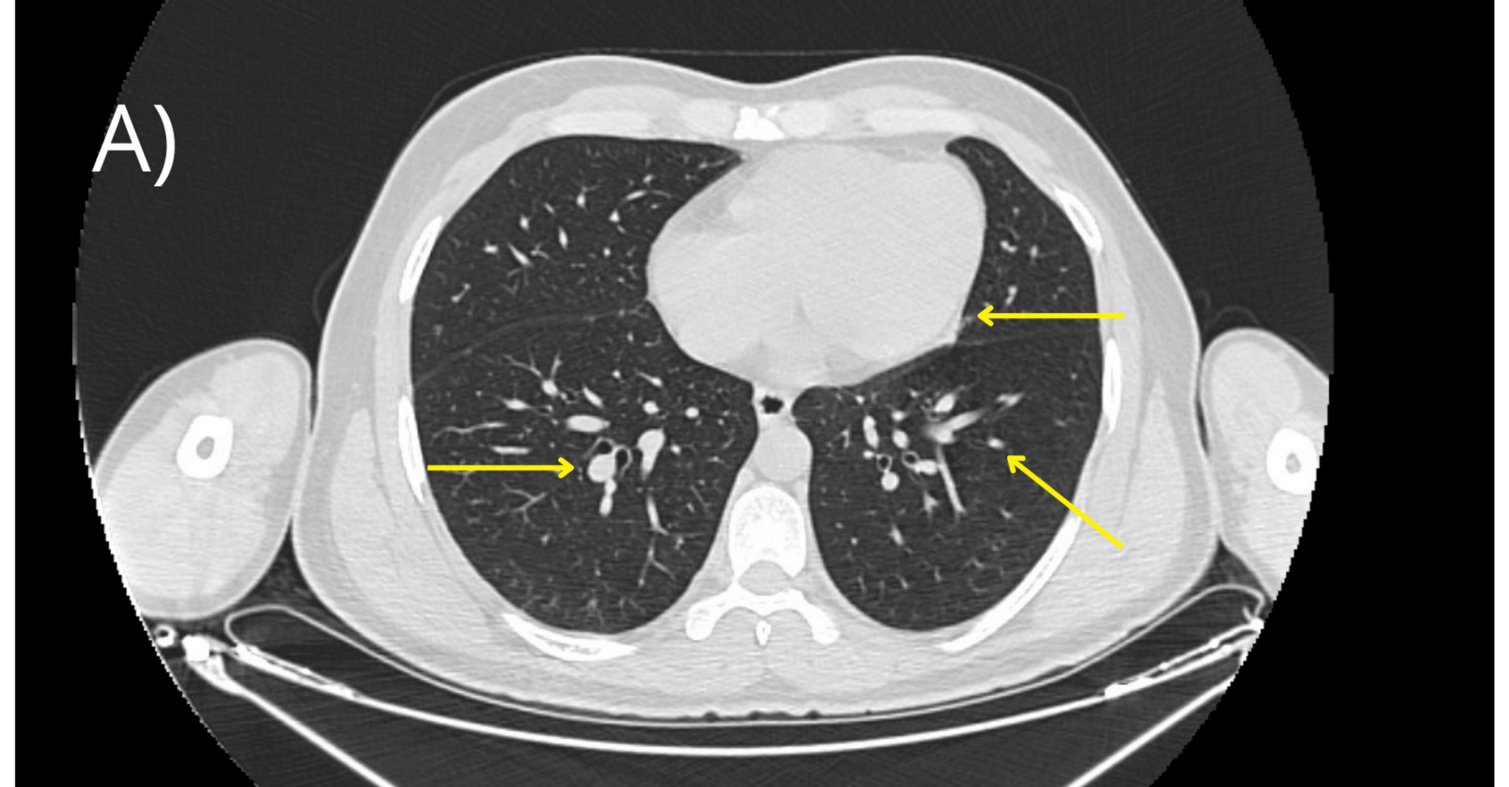
Follow-up contrast-enhanced thoracic CT showing decreased size of pulmonary lesions.

At three months, follow-up contrast-enhanced cervical and thoracic computed tomography (Figure 3) demonstrated a reduction in the size of the pulmonary lesions, with persistence of thrombotic involvement.

**Figure 4:**
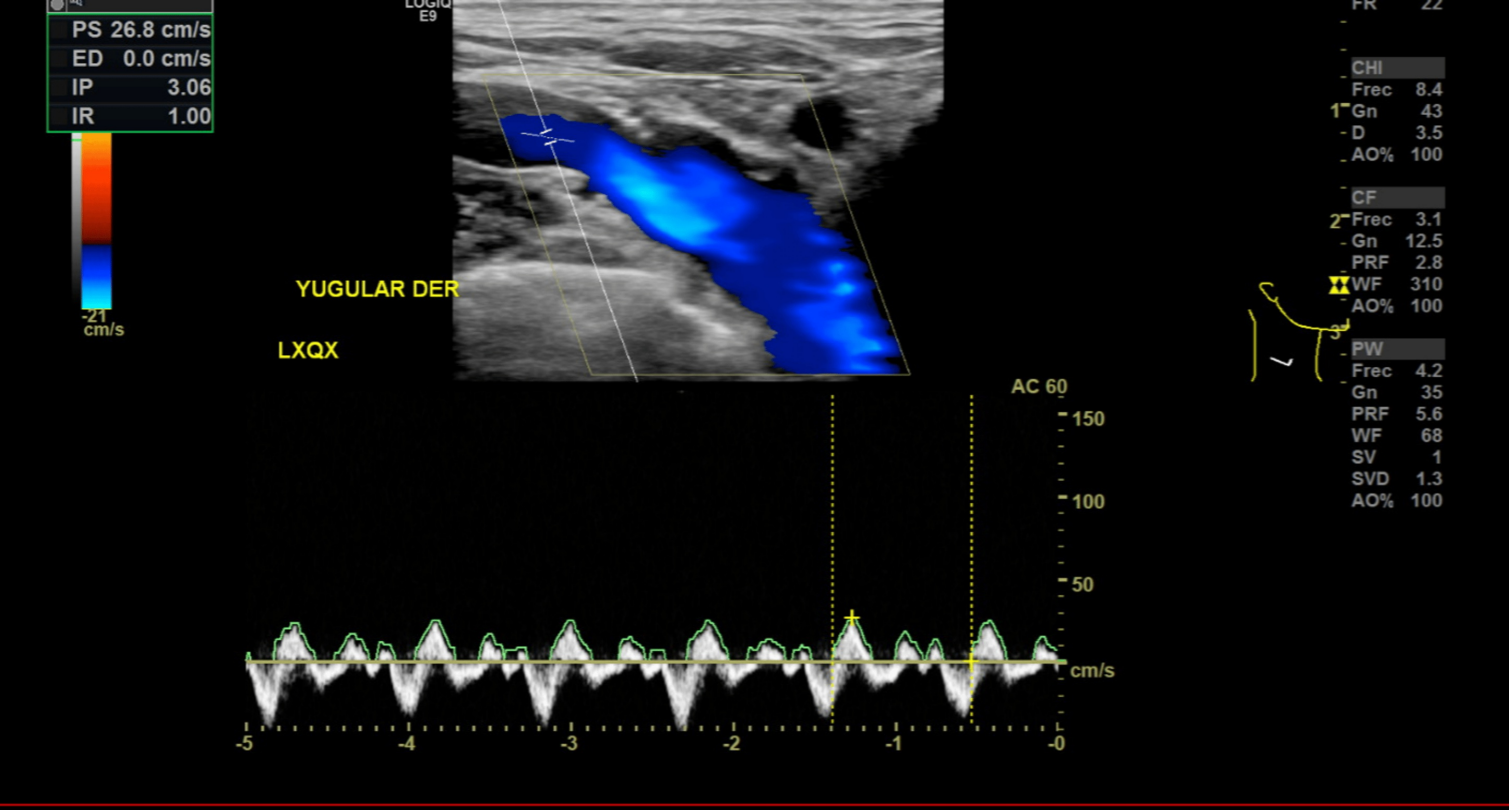
Follow-up Doppler ultrasound of the right jugular vein showing no thrombotic involvement and adequate blood flow.

At six months, anticoagulation was discontinued after Doppler ultrasound (Figure 4) confirmed resolution of jugular and carotid thrombosis, with no residual collections or lesions. The patient was discharged with mild residual dysphonia and cough, with recommendations for vocal hygiene exercises and routine oral care. Overall, the prognosis was favorable in terms of functional recovery and quality of life.

## Discussion

Lemierre syndrome is characterized by a biphasic evolution that begins with an infection of the head and neck and may progress to septic thrombophlebitis of the internal jugular vein and sepsis, often with systemic involvement [1-4]. In this case, a typical pharyngeal infection was not documented; however, an odontogenic source was identified following a dental extraction, with extension into the deep cervical spaces, and Ludwig’s angina was considered in the differential diagnosis. This finding aligns with recent reports recognizing non-oropharyngeal foci as possible sources of the syndrome, thereby expanding its clinical spectrum [2].

The pattern of dissemination observed reflects the classical behavior of the disease. The presence of extensive thrombosis of the jugular venous system associated with septic pulmonary embolization corresponds to the hematogenous spread mechanism described in most cases [2,3,6]. Likewise, the bilateral pulmonary lesions mirror the typical pattern of predominant pulmonary involvement [2,3,8]. However, the absence of significant respiratory compromise and the patient’s hemodynamic stability contrast with prior reports, where extensive pulmonary involvement is usually associated with respiratory failure and severe systemic manifestations, including sepsis or septic shock [2-4].

Cohort studies have shown that even in the presence of jugular thrombosis and pulmonary embolization, clinical evolution may be heterogeneous: while some patients remain clinically stable, others progress to severe sepsis or septic shock [7,10]. Similarly, cases with comparable radiological thrombotic burden have been reported to evolve toward multiple organ failure, indicating that thrombus extent alone may not be a reliable predictor of clinical severity. Taken together, these findings support the concept that Lemierre syndrome represents a clinical continuum rather than a uniform entity, influenced by host factors, bacterial virulence, and timing of treatment initiation [4,7].

Consistent with this, our patient had a favorable course, without progression of thrombotic events or development of new septic foci, in contrast to reports where such complications may occur early [7]. Persistent dysphonia secondary to vocal cord paresis is interpreted as a local sequela within the spectrum of described complications [2,4].

From an anatomical perspective, the venous extension observed exceeds the usual presentation. In addition to internal jugular vein thrombosis, involvement of the jugular bulb, sigmoid sinus, and right subclavian vein was documented. The literature describes that thrombosis may progress from initial endothelial alterations to complete occlusion, with possible contiguous extension to adjacent venous structures [9,10]. The findings in this case are in keeping with these propagation mechanisms, although they represent a less frequent variant. Despite this, no major intracranial complications were observed, such as cavernous sinus thrombosis or ischemic cerebrovascular events, which are associated with a worse prognosis [2,9].

From a microbiological standpoint, the syndrome is primarily associated with *Fusobacterium necrophorum*, although the involvement of multiple microorganisms is common [2,3,6]. In this case, no pathogen was isolated, a finding well described in the literature, particularly following prior antibiotic administration or due to the inherent difficulties in culturing anaerobes [2,6]. Prior exposure to clindamycin could explain this result; however, the persistence of purulent material and clinical progression suggest a polymicrobial process with possible insufficient initial coverage. This phenomenon is well documented, as the isolation of anaerobes may be delayed or unsuccessful, particularly when antibiotics have been initiated prior to sample collection or when culture conditions are suboptimal [2,3]. In contemporary series, a relevant proportion of patients present with negative or polymicrobial cultures, supporting both the technical limitations of pathogen recovery and the potential role of coinfections [6]. In this context, negative cultures do not exclude the diagnosis and should be interpreted within the overall clinical picture.

The lack of resolution with clindamycin, despite its activity against anaerobes, underscores the need for broad-spectrum regimens in deep cervicofacial infections with risk of dissemination. Additionally, persistence of the abscess may be explained by limited antibiotic penetration into the septic thrombus, making surgical drainage a necessary component of treatment [3]. Escalation to piperacillin/tazobactam is consistent with current recommendations, particularly in the context of potential β-lactamase-producing organisms [3,6].

Regarding thrombosis management, anticoagulation remains controversial, with non-standardized indications and limited evidence regarding its impact on clinical outcomes. Several studies have shown conflicting results, without consistently demonstrating benefits in vascular recanalization or mortality [1,10]. Nevertheless, its use is relatively common in clinical practice, particularly in more severe scenarios, such as extensive thrombosis, thrombus progression despite antibiotic therapy, involvement of intracranial venous sinuses, or persistent septic embolization [2,8,10]. Some analyses suggest that anticoagulation may be associated with a lower incidence of new thromboembolic or septic complications; however, these findings derive from observational studies with a high risk of bias and do not allow causal inference [7,10]. Consequently, most current recommendations emphasize individualized decision-making, balancing thrombotic and hemorrhagic risks in each patient [2,4]. In general terms, available evidence does not support the routine use of anticoagulation but rather a selective approach in high-risk scenarios. In this case, anticoagulation with enoxaparin was considered justified due to the extensive thrombotic involvement and proximity to intracranial territories [8,10]. Resolution of thrombosis at six months reflects a favorable outcome within the implemented therapeutic approach, although it does not allow for a definitive causal relationship with anticoagulation [8,10].

## Conclusions

In conclusion, this case illustrates a presentation within the spectrum of Lemierre syndrome with notable features, including an odontogenic origin, extensive venous involvement, and the absence of significant systemic compromise. Importantly, the substantial thrombotic burden observed on imaging did not correlate with a severe clinical course, supporting a dissociation between radiological findings and systemic severity and underscoring the variable clinical spectrum of the disease.

These findings emphasize the need to consider Lemierre syndrome in deep cervicofacial infections, particularly those of odontogenic origin, even in the absence of a pharyngeal antecedent. From a practical standpoint, this case underscores the value of early imaging in patients with atypical clinical progression, as well as prompt multidisciplinary management to optimize diagnosis and guide timely therapeutic decisions.
